# The genetic basis of obesity-associated type 2 diabetes (diabesity) in polygenic mouse models

**DOI:** 10.1007/s00335-014-9514-2

**Published:** 2014-04-22

**Authors:** Hans-Georg Joost, Annette Schürmann

**Affiliations:** 1 Department of Pharmacology, Department of Experimental Diabetology, German Institute of Human Nutrition Potsdam-Rehbrücke, Arthur-Scheunert-Allee 114-116, 14558 Nuthetal, Germany; 2 German Center of Diabetes Research, Neuherberg, Germany

## Abstract

Obesity-associated diabetes (“diabesity”) in mouse strains is characterized by severe insulin resistance, hyperglycaemia and progressive failure, and loss of beta cells. This condition is observed in inbred obese mouse strains such as the New Zealand Obese (NZO/HlLt and NZO/HlBomDife) or the TALLYHO/JngJ mouse. In lean strains such as C57BLKS/J, BTBR T+tf/J or DBA/2 J carrying diabetes susceptibility genes (“diabetes susceptible” background), it can be induced by introgression of the obesity-causing mutations *Lep* *<ob>* (*ob*) or *Lepr* *<db>* (*db*). Outcross populations of these models have been employed in the genome-wide search for mouse diabetes genes, and have led to positional cloning of the strong candidates *Pctp*, *Tbc1d1*, *Zfp69*, and *Ifi202b* (NZO-derived obesity) and *Sorcs1,* *Lisch-like*, *Tomosyn-2, App, Tsc2,* and *Ube2l6* (obesity caused by the *ob* or *db* mutation). Some of these genes have been shown to play a role in the regulation of the human glucose or lipid metabolism. Thus, dissection of the genetic basis of obesity and diabetes in mouse models can identify regulatory mechanisms that are relevant for the human disease.

## Introduction: rationale for the use of mouse models in the investigation of the metabolic syndrome

The human metabolic syndrome is a complex combination of the traits visceral obesity, dyslipoproteinemia, hypertension, and insulin resistance which have life-shortening consequences such as type 2 diabetes and atherosclerosis. Inbred mouse strains, e.g., the New Zealand Obese mouse (NZO/HlLt and NZO/HlBomDife; NZO), the C57KS/J-*db/db* mouse, or the TALLYHO/JngJ (TALLYHO; TH) mouse, present a similar syndrome of obesity-associated insulin resistance, beta-cell failure, and ultimately chronic hyperglycaemia. For this type 2 diabetes-like condition, the term diabesity was coined, and it appears reasonable to assume that many, if not all, pathogenetic mechanisms leading to hyperglycaemia are similar in mice and humans. Therefore, mouse models have been widely used to investigate the pathogenesis of the metabolic syndrome, and particular efforts have been made to identify the gene variants that are responsible for the traits obesity and diabetes (beta cell loss). Because of their much higher diabetes susceptibility, male mice were used exclusively in almost all studies.

A second advantage of the mouse model is that breeding experiments are possible that lead to the localization of disease genes. By that approach, a mutant gene responsible for extreme obesity in the *ob/ob* mouse was cloned and shown to encode leptin, an anorexigenic peptide secreted from the adipocyte (Zhang et al. 1994). This landmark discovery was soon followed by identification of other diabesity genes in monogenic mutant strains, namely the *db* (leptin receptor), agouti yellow, tubby, fat, and mahogany mutations (Friedman 1997; Leibel et al. 1997). These findings led to the elucidation of the neuroendocrine regulation of hunger and satiety controlled by peptides such as MSH and NPY (Woods and D’Alessio 2008). Subsequently, it was shown that mutations of human orthologs (*LEP, LEPR*) or of functionally associated genes (*POMC*, *MCR4*) produced phenotypes comparable with that of the respective mouse models (Clement et al. 1998; Santini et al. 2009).

The third advantage of the mouse model is that after identification of a candidate gene, direct genetic evidence for its involvement in a pathophysiology can be obtained in mice, but very rarely in humans. Thus, inbred mouse models are ideally suited for the investigation of the obesity-associated diabetes. However, the genetic homogeneity of the inbred strains is not only an advantage, it also limits their potential. Individuals of an inbred mouse line are genetically identical, and it cannot be expected that a single strain carries more than a small portion of all relevant gene variants. Currently, more than 2000 mouse QTL for different traits have been identified in crosses between inbred stains, but only about 1 % has been characterized on molecular level (Flint et al. 2005). Thus, more than one model and new resources, e.g., systems biology may be required for a complete genetic analysis of complex traits. Previous and ongoing research supports the view that the combination of individual genomes—by intercross of inbred strains and by the generation of congenic lines—will reveal effects of many more genes and gene interactions than can be observed in a single inbred strain. Because the cross-breeding experiments are time consuming and expensive, selecting the “right” models of the obesity-associated diabetes is of crucial importance (Leiter 2009). Another advantage of mouse studies in comparison to human studies is the ability to control the environment and to investigate effects of diets, exercise, and intestinal microbiota.

## Polygenic basis of “diabesity” in mice: the interaction of obesity and diabetes genes

Obesity-associated diabetes (“diabesity”) is due to interaction of genes causing obesity with diabetes genes. This conclusion is based on findings indicating that obesity is a necessary but not sufficient condition for the type 2 diabetes-like hyperglycaemia: Obese mice are insulin resistant and therefore more or less glucose intolerant, but in some strains such as C57BL/6J-*ob/ob*, insulin resistance is compensated by hyperinsulinemia and beta cell hyperplasia, and plasma glucose is only moderately elevated. Other models such as C57BLKS/J-*db/db* and NZO present overt diabetes mellitus as defined by a threshold of 16.6 mM (300 mg/dl) plasma glucose (Leiter et al. 1998); mice crossing this threshold usually exhibit progressive failure and subsequent apoptosis of beta cells. This type 2 diabetes-like condition is not due to the obesity-causing gene variants but to other genes in the genetic background of the strain, which cause obesity-associated diabetes. The severe and early onsetting diabetes of the C57BLKS/J-db/db strain is due to the C57BLKS/J background, since mice carrying the *db* mutation on the C57BL/6J background are not diabetic (Stoehr et al. 2000). Conversely, C57BL/6J-*ob/ob* mice are normoglycemic, whereas introgression of the *ob* mutation into the C57BLKS/J background produced a severely diabetic strain (Coleman 1978). Furthermore, it has been shown that in crosses of lean, normoglycaemic strains with diabetic strains the lean strain can introduce variants that markedly aggravate the diabetic phenotype (Leiter et al. 1998; Plum et al. 2000).

Thus, lean mouse strains can be diabetes-susceptible (e.g. C57BLKS/J, BTBR T+tf/J, NON/Lt) or diabetes-resistant (e.g. C57BL/6J, 129/SvJ), as defined by the phenotype of the strain when an obesity-causing mutation has been introduced (Herberg and Leiter 2001). Consequently, lean strains can be valuable models for identification, and considerable efforts have been made to identify the genes responsible for this metabolic dichotomy. Furthermore, there is a second conclusion from the above-described findings: since obesity is required for the penetrance of diabetes genes, their identification by random or targeted mutagenesis requires introduction of obesity genes in a “reporter” cross.

## Experimental strategy for identification of mouse genes causing obesity and diabetes

The conventional strategy for identification of mouse disease genes is the genome-wide linkage analysis of outcross populations. This approach led to numerous susceptibility loci (quantitative trait loci, QTL) of obesity and diabetes-related traits such as body weight, fat mass, plasma glucose or insulin levels, and beta cell mass (for review and meta-analysis of these studies see Wuschke et al. 2007; Schmidt et al. 2008). So far, only few responsible gene variants have been identified in these QTL. Because of the complex interaction of genes, a particular locus may be detected in one intercross, and may be hidden in another. Conventional strategy of positional cloning then requires the introgression of a locus in a different strain, or into the lean, non-diabetic breeding partner, and subsequently the definition of a critical chromosomal region by further inbreeding of the subcongenic line. When available, chromosome substitution strains and congenic lines generated thereof allow an analysis of the architecture of a complex QTL, and a refinement of the QTL into very small segments (Nadeau et al. 2000; Yazbek et al. 2011). The critical region can then be investigated by sequence analysis and functional characterization of its genes.

This strategy can considerably reduce the number of candidates, because it safely excludes genes not present in the critical region. In our hands, the generation of subcongenic strains was successful in the positional cloning of the obesity genes *Tbc1d1* (Chadt et al. 2008) and *Ifi202b* (Vogel et al. 2012) and the diabetes gene *Zfp69* (Scherneck, et al. 2009). However, the strategy is not always feasible because the phenotype may be lost by the isolation of the responsible gene on a different background. In these cases, technologies such as mRNA profiling that allow the investigation of thousands of genes can be used for a search in the whole QTL. Furthermore, such a systems biology approach combining genetic (mapping) and functional (gene expression) data can analyze interactions between QTL and leads not only to plausible candidates but also to a mechanistic understanding of the pathogenesis (Lusis et al. 2008; Ferrara et al. 2008; Keller et al. 2008; Wang et al. 2011; Davis et al. 2012). Metabolomics data may also be combined with the genetic information and can lead to candidates as well as to mechanistic insight. In addition, sequence comparisons of whole genome are feasible and may lead to the identification of a responsible gene, if an assay is available that proves loss or gain of its function. Refining of a locus by mapping haplotype blocks is a possibility but may lead to wrong information when the causal mutation is “younger” than the haplotype block it is located in.

Obviously, outcross populations of only two strains cannot reflect the full complexity of polygenic traits and diseases. Thus, the collaborative cross was established which is a large panel of recombinant inbred strains derived from a set of 8 genetically diverse founder strains (Churchill and Complex Trait Consortium 2004). This panel might also help to elucidate the genetic basis of obesity, insulin resistance, and diabetes. However, it should be noted that the strains of the panel might be too lean to show failure and apoptosis of beta cells. More recently, Parks et al. (2013) performed a systems genetic study comparing more than 100 inbred mouse strains: genome-wide genotyping (GWAS) of these strains identified 11 genes that were significantly associated with obesity (e.g. *Sfrp5, Chrebp, Tmem160*), and indicated considerable overlap with loci identified in human GWAS.

For the identification of obesity genes, genome-wide mutagenesis (*Drosophila melanogaster*) and siRNA approaches (*Cenorhabditis elegans*) can be helpful. *G*enes that were found to be associated with adiposity in these studies can be validated in the mouse. By this approach, we identified a variant of the cholesterol transporter *Abcg1* from NZO which increased adiposity (Buchmann et al. 2007).

## Polygenic mouse models of the metabolic syndrome

### The New Zealand obese mouse

New Zealand obese (NZO) mice present a syndrome of morbid obesity, insulin resistance, hypertension, and hypercholesterolemia which resembles the human metabolic syndrome (Bielschowsky and Bielschowsky 1953; Herberg and Coleman 1977; Ortlepp et al. 2000; Kluge et al. 2012). Obesity is due to a moderately increased food intake and reduced energy expenditure with reduced body temperature (Koza et al. 2004; Jürgens et al. 2006). As a consequence of the syndrome, male NZO mice develop type 2 like diabetes characterized by marked hyperglycaemia and hyperinsulinemia at earlier age (8–12 weeks), and later on by low serum insulin levels associated with beta-cell destruction (Crofford and Davis 1965; Leiter et al. 1998; Jürgens et al. 2006). The syndrome has a polygenic basis, and outcross progeny of the strain as well as generation of subcongenic lines has previously been used for identification of genes associated with adiposity, hypercholesterolemia, and hyperglycaemia (Leiter et al. 1998; Reifsnyder et al. 2000; Plum et al. 2000; Kluge et al. 2000; Taylor et al. 2001; Reifsnyder and Leiter 2002; Plum et al. 2002; Giesen et al. 2003; Vogel et al. 2009; Vogel et al. 2012).

### Strains rendered obese by introgression of mutations causing monogenic obesity

The loss-of-function mutations in the leptin (*Lep <ob>;* ob) and in the leptin receptor gene (*Lepr <db>;* db) cause morbid obesity, and depending on the background, may cause severe hyperglycaemia. As was outlined above, lean strains may carry diabetogenic or diabetes-suppressing alleles which produce a phenotype only after introgression of the adipogenic mutations. Thus, several crosses were performed with lean diabetes-susceptible strains (C57BLKS/J, BTBR, DBA/2J, BALB/c) which were crossed with the diabetes-resistant C57BL/6J (B6) strain. Breeding pairs for generation of F2 or backcross populations had to be heterozygous for the *ob* or *db* mutation, and homozygous progeny was analyzed. This strategy led to identification of the candidate genes *Sorcs1* (BTBR; Clee et al. 2006), *Lisch-like* (DBA/2J; Dokmanovic-Chouinard et al. 2008), *Tomosyn-2* (BTBR T+tf/J; Bhatnagar et al. 2011), and *App* (BTBR T+tf/J; Tu et al. 2012) which are presumably responsible for the beta cell phenotype of the progeny. In addition, candidate genes causing fatty liver (*Tsc2*; Wang et al. 2012) or suppressing obesity (*Ube2l6;* Marcelin et al. 2013) were identified in crosses with BALB/c, respectively. An advantage of this model is that obesity in the *ob/ob* or *db/db* progeny is less variable than in outcross populations of polygenic strains. A disadvantage is that chromosomal regions close to the *ob* or *db* locus co-segregate with the mutations and cannot be studied in these crosses. Another disadvantage is that the complete absence of leptin signaling produces a unique genetic background which is probably not descriptive of human diabesity where the leptin and leptin receptor axis is at least partially intact.

### The TALLYHO mouse

The TALLYHO/JngJ (TH) mouse strain was generated by selective breeding for hyperglycemia from an outbred colony of mice (Kim and Saxton 2012). TH mice are obese, hyperinsulinemic, and hyperlipidemic. Similar to NZO, male TH mice develop severe hyperglycaemia (400 mg/dl at 16 weeks of age). Obesity is less pronounced than in NZO (35 g at weak 16), and, in contrast to NZO, blood pressure of TH mice is normal. Analysis of F2 backcross and intercross populations revealed numerous loci and epistatic interactions accounting for the hyperglycaemia, obesity, and hypertriglyceridemia in TH mice (Kim et al. 2001; Kim et al. 2005; Stewart et al. 2010). Congenic mice carrying the Chr 6 locus on the B6 background confirmed the QTL for obesity (Kim et al. 2005) and were further analyzed (Stewart et al. 2012), but have not yet led to a candidate gene. A comparison of the TH strain with a diabesity model derived from the NZO and NON strains (NONcNZO10Lt/J) revealed remarkable similarities in spite of the clear genetic differences (Leiter et al. 2013).

### Lean strains rendered obese by dietary intervention

Moderate obesity can be induced in some lean strains by feeding a high fat and/or sugar containing diet instead of chow. Under these conditions, insulin resistance, hyperinsulinemia, and impaired glucose tolerance develops. Numerous crosses between lean strains such as C57BL/6J and C3H/HeJ (Toye et al. 2005; Li et al. 2012) kept on an adipogenic diet were performed in order to map the genes responsible for obesity and glucose intolerance (reviewed by Wuschke et al. 2007; Schmidt et al. 2008). Furthermore, several other crosses, e.g., between B6 and CAST mice, noted for its low fat intake and preference for carbohydrates, have been generated to clarify the pathogenesis of increased energy intake and obesity, and were characterized on different adipogenic or atherogenic diets (Smith Richards et al. 2002; Estrada-Smith et al. 2004; Farber et al. 2009). It has to be noted that diabetes (severe hyperglycaemia >300 mg/dL), and beta cell disruption does not occur in these models. Thus, identification of the genes responsible for the diabetic phenotype may require the models of morbid obesity described above.

## Candidate genes associated with obesity and diabetes that were identified in NZO and NZO-derived outcross populations

**Fig. 1:**
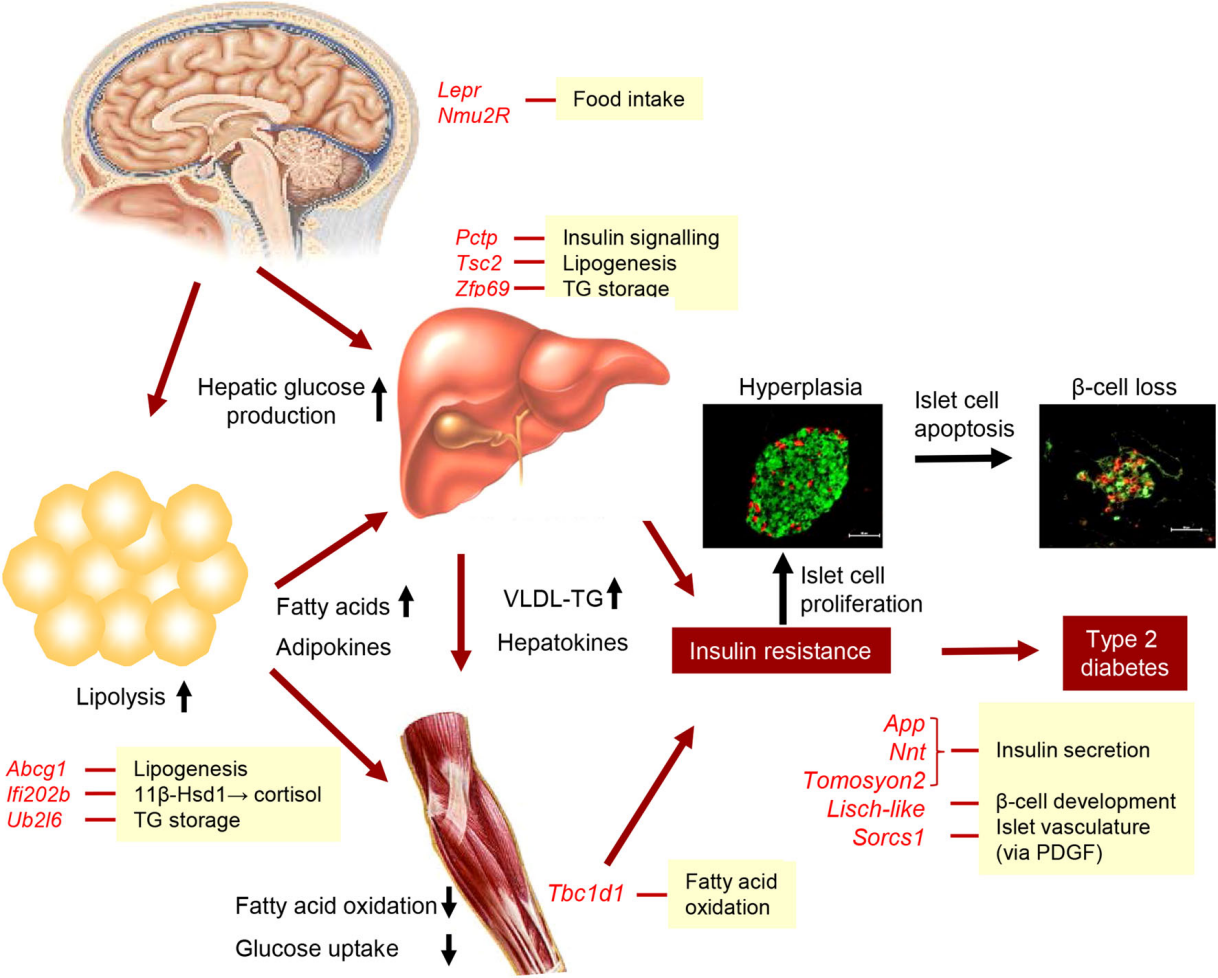
Pathophysiology of insulin resistance and type 2 diabetes with beta-cell failure, and site of action of candidate genes identified by positional cloning (highlighted in *red*). In response to increased food intake and/or reduced energy expenditure, obesity develops as a major cause of insulin resistance. Due to impaired signaling, insulin fails to adequately suppress lipolysis in adipose tissue and release of glucose and VLDL from the liver. Furthermore, fatty acid oxidation and glucose uptake by the skeletal muscle are reduced, aggravating insulin resistance and glucose intolerance. In the insulin resistant state, pancreatic islets initially compensate by hyperplasia. However, in the presence of diabetes genes apoptosis results in beta-cell loss and the development of manifest type 2 diabetes

All adipogenic and diabetogenic gene variants identified in outcross populations of obese mice are listed in Table 1, and their putative sites of action are shown in Fig. 1.

**Table 1:** Adipogenic and diabetogenic gene variants identified in outcross populations of obese mice

Gene symbol	Gene name/function	Variant	Trait associated with variant	Obesity induced by	Strains with variant	Reference
*Pctp*	Phosphatidylcholine transfer protein	1 amino acid substitution, reduced activity	Serum insulin	NZO background	NZO, NZB	Pan et al. (2006a, b), Ersoy et al. (2013)
*Tbc1d1*	Rab-GTPase activating protein	Deletion/frameshift and truncation of protein	Accumulation of body fat	NZO background	SJL	Chad et al. (2008)
*Zfp69*	Zinc finger domain transcription factor	Premature polyadenylation caused by retrotransposon	Plasma glucose and insulin, hepatic fat	NZO background	NZO, C57BL/6J	Scherneck et al. (2009)
*Ifi202b*	Interferon-activated 202b	Deletion of promoter and exon 1, no expression	11ß-HSD expression, adiposity	NZO background	C57BL/6J	Vogel et al. (2012)
*Lepr*	Leptin receptor	4 coding SNPs (V541I, V651I, A720T, T1044I)	Food intake	NZO background	NZO, NZB	Igel et al. (1997), Kluge et al. (2000)
*Abcg1*	ATP-binding cassette transporter G1	Insertion of LXR element in intron 2, increased expression of *Abcg1*	Accumulation of body fat	NZO background	NZO, NZB	Buchmann et al. (2007)
*Nmur2*	Neuromedin U receptor 2	2 coding SNPs (V190 M, I202 M), reduced activity	Food intake	NZO background	NZO, NZB	Schmolz et al. (2007)
*Sorcs1*	Sortilin-related VPS10 domain containing receptor	Reduced expression and 3 coding SNPs (T52I, S1140F, S1150P)	beta-cell disruption, development of islet vasculature	*ob/ob*	BTBR T+tf/J	Clee et al. (2006)
*Lisch-like*	Similar to Lisch7/Lsr (lipolysis stimulated receptor).	Reduced expression and 2 coding SNPs (T587A, A647 V)	beta-cell disruption	*ob/ob*	DBA/2 J	Dokmanovic-Chouinard et al. (2008)
*Tomosyn-2*	Syntaxin-binding protein 5-like	Increased expression and coding SNP (S912L)	Reduced insulin secretion	*ob/ob*	BTBR T+tf/J	Bhatnagar et al. (2011)
*Tsc2*	Tuberous sclerosis complex 2	Coding SNP, reduced activity inhibiting lipogenesis	Hepatic steatosis	*ob/ob*	BTBR T+tf/J	Wang et al. (2012)
*App*	Amyloid precursor protein	Increased expression in beta cells	Insulin secretion	*ob/ob*	BTBR T+tf/J	Tu et al. (2012)
*Ube2l6*	Ubiquitin-conjugating enzyme E2L8	Coding SNP (D29Y) and suppressed expression	Fat mass, reduced lipid synthesis	*ob/ob*	BALB/c	Marcelin et al. (2013)
*Slc35b4*	Solute carrier family 35 member B4, transporter of xylose in Golgi	Increased expression in liver	Reduced production of glucose, increased insulin sensitivity	Diet	A/J	Yazbek et al. (2011)
*Apcs*	Amyloid P component	Increased expression in liver	Fasting glucose	Diet	C3H/HeJ	Li et al. (2012)
*Nnt*	Nicotinamide nucleotide transhydrogenase	Deletion of 5 exons, absence of protein	Reduced glucose-stimulated insulin secretion	No obesity	C57BL/6J	Toye et al. (2005)

### Phosphatidyl choline transfer protein (*Pctp*)

The characterization of an outcross population of NZO/HI with NON/Lt mice led to the identification of a major QTL for hyperglycaemia and hypoinsulinemia on Chr 11 *(Nidd3)* (Leiter et al. 1998), and to the finding that NZO mice exhibit an impaired phosphatidyl choline metabolism (Pan et al. 2006a). The QTL comprises the gene encoding phosphatidyl choline transfer protein (PC-TP) which is a specific phosphatidyl choline-binding protein and regulates hepatic lipid metabolism. Sequencing and functional studies indicated that NZO carries an inactive variant (R120H) which could be responsible for the diabetogenic effect of *Nidd3* (Pan et al. 2006b). Recently, it was reported that siRNA-mediated knockdown, genetic ablation of *Pctp* or chemical inhibition of PC-TP increases insulin sensitivity by enhancing IRS2 activation (Ersoy et al. 2013).

### TBC-domain protein 1 (*Tbc1d1*)

By genome-wide linkage analysis of an outcross population of NZO/HIBomDife with lean SJL/J mice, a major QTL for body weight was identified on chromosome 5 (Kluge et al. 2000). Additional outcross experiments suggested that the responsible variant allele was an obesity suppressor unique for SJL. Characterisation of recombinant congenic lines carrying the variant on a C57BL/6J background defined a critical region which was characterized by sequencing and gene expression profiling. By this approach, it was shown that SJL mice carry a loss-of-function variant of the RabGAP *Tbc1d1* generated by a 7 bp in-frame deletion which produces a truncated protein (Chadt et al. 2008). Introgression of the variant into NZO reduced body weight and suppressed the development of diabetes. Knockdown of endogenous *Tbc1d1* in C2C12 muscle cells increased palmitate uptake and oxidation, and reduced glucose oxidation (Chadt et al. 2008). In C57BL/6J mice, the variant reduced body weight, enhanced fat oxidation, and reduced glucose oxidation (Chadt et al. 2008; Dokas et al. 2013). Thus, adiposity and diabetes in obese mice are modified by disruption of *Tbc1d1* through a metabolic shift from glucose to fat oxidation. This mechanism may also explain the association of the human R125W *TBC1D1* variant with obesity in Utah (Stone et al. 2006) and French families (Meyre et al. 2008).

### Zinc finger protein 69 *(Zfp69)*

In the backcross population of NZO/HIBomDife with SJL/J, an allele causing acceleration and aggravation of the diabetes was mapped to distal chromosome 4 (Plum et al. 2000). The diabetogenic effect of the QTL was markedly enhanced by NZO chromosome 5 (*Tbc1d1*) and by a high-fat diet (Plum et al. 2002). Interval-specific congenic introgression of SJL into diabetes-resistant C57BL/6J, and subsequent reporter cross with NZO, led to the identification of a critical interval of Chr 4 (2.1 Mbp) conferring the diabetic phenotype (Scherneck et al. 2009). Analysis of the 10 genes in the critical interval by sequencing and qRT-PCR revealed a striking allelic variance of the zinc finger domain transcription factor 69 (*Zfp69*); in NZO and C57BL/6J, mRNA of *Zfp69* was nearly undetectable. This difference was due to the presence of a retroviral transposon (IAPLTR1a) in intron 3 of NZO and C57BL/6J which caused a premature polyadenylation of the *Zfp69* mRNA (Scherneck et al. 2009). The transposon disrupts the gene by formation of a truncated mRNA that lacked the coding sequence for the KRAB and Znf-C2H2 domains of *Zfp69*. In contrast, the diabetogenic alleles from SJL, NON, and NZB lacked the transposon and generated a normal mRNA. When combined with the C57BL/6J-*ob/ob* background, the diabetogenic SJL allele of *Zfp69* produced hyperglycaemia, reduced gonadal fat, and increased plasma and liver triglycerides. mRNA levels of the human orthologue of *Zfp69* and *ZNF642* were significantly increased in adipose tissue from patients with type 2 diabetes. Thus, *Zfp69* is the most likely candidate for the diabetogenic effect of *Nidd/SJL*. Expression of the transcription factor in adipose tissue may play a role in the pathogenesis of type 2 diabetes. In addition, retrotransposon IAPLTR1a appears to contribute substantially to the genetic heterogeneity of mouse strains, since it produced aberrant mRNA species of 7 other genes (Scherneck et al. 2009).

### Interferon-activated gene 202b (*Ifi202b*)

In an outcross F2 population of NZO/HIBomDife with the diabetes-resistant C57BL/6J strain, a major QTL responsible for obesity and hyperglycaemia was identified on Chr 1 (Vogel et al. 2009). The QTL is particularly important, since it interacted with the diabetogenic *Zfp69* allele; the two loci accounted for almost all of the diabetes in the cross experiments NZO × B6.Cg-Nidd/SJL designed to identify the diabetogenic gene in Nidd/SJL (Scherneck et al. unpublished data). By introgression of the chromosomal segment into B6, a critical region harboring approximately 30 genes was defined. Expression profiling identified a major allelic difference: mRNA of the interferon-activated gene 202b (*Ifi202b*) was generated in NZO, but was undetectable in C57BL/6J. This difference was due to a microdeletion of approximately 30 Mbp in B6 which comprised exon 1 and the 5’-flanking region of *Ifi202b* (Vogel et al. 2012). Whole genome differential mRNA profiling indicated that the disruption of *Ifi202b* reduced the expression of 17β-hydroxysteroid dehydrogenase in adipose tissue. Overexpression and knockdown of *Ifi202b* in 3T3-L1 preadipocytes indeed induced or repressed expression of 17β-HSD. Since 17β-HSD has previously been associated with obesity, *Ifi202b* is a most plausible candidate gene contributing to the effects of the QTL on Chr 1. It is worth noting that the *Ifi202b* locus in addition to adiposity also modulates behavioral traits, and that it maps to a QTL hotspot of behavioral phenotypes identified in 32 different outcross populations (Vogel et al. 2013).

### Leptin receptor *(Lepr)*

Sequencing revealed that NZO mice carry a leptin receptor variant with 4 amino acid exchanges including two non-conservative substitutions (A720T, T1044I) (Igel et al. 1997). There is suggestive evidence that this variant contributes to the obesity of the NZO strain, since the *Lepr* locus appeared to enhance the effect of the QTL *Nob1* (later identified as *Tbc1d1*) on body weight and serum insulin in female (SJLxNZO)NZO backcross mice (Kluge et al. 2000). Functional studies of the receptor variant expressed in COS-7 cells indicated an only small reduction of its signaling potential (Kluge et al. 2000). The contribution of the receptor variant to the obesity syndrome of the NZO mouse appears to depend on other adipogenic alleles, since the variant is also present in the related New Zealand Black (NZB) strain which shows neither obesity nor insulin resistance. Furthermore, it has been suggested that hyperphagia of NZO mice could be a consequence of impaired brain uptake of leptin (Hileman et al. 2002).

### ATP-binding cassette transporter G1 (*Abcg1*)

Association of the ATP-binding cassette transporter G1 (ABCG1) with obesity was discovered in a screening approach that compared data from the *Drosophila melanogaster* and mouse genomes (Buchmann et al. 2007). In a random mutagenesis screen, *Drosophila* genes involved in triglyceride storage were identified, and their mouse orthologs that mapped to obesity QTL were further analyzed. Overexpression of the *Drosophila* orthologue of *Abcg1* generated lines of flies with increased triglyceride stores. *Abcg1* is located in a suggestive obesity QTL on proximal Chr 17, and NZO mice carry an insertion of multiple LXR-responsive elements that was associated with higher expression of the gene in white adipose tissue. Targeted disruption of *Abcg1* in mice reduced adipose tissue depots, decreased the size of the adipocytes, and prevented high-fat diet-induced insulin resistance and fatty liver. Furthermore, mRNA levels of the cholesterol-regulated transcription factor LXRα and of its downstream target ABCA1 were increased in adipose tissue of male *Abcg1* ^*−/−*^ mice. Thus, it has been suggested that ABCG1 regulates triglyceride storage by controlling intracellular cholesterol as a key regulator of gene expression in the adipocyte (Buchmann et al. 2007).

### Neuromedin U receptor 2 (*Nmur2*)

Sequencing of the known genes involved in the regulation of energy balance led to the identification of a variant neuromedin U receptor 2 in NZO carrying two amino acid substitutions (V190M and I202M) (Schmolz et al. 2007). The *Nmur2* gene is located in a suggestive obesity QTL on chromosome 11, distal to the diabetes QTL *Nidd3*. Neuromedin U exerts an anorexigenic effect on meal frequency that is blunted in NZO as compared with lean C57BL/6J mice (15 vs. 60 % reduction in C57BL/6J). Transfection of HEK293 cells with wild-type and variant *Nmur2* cDNA indicated a reduced signaling potential of the variant receptor (EC_50_ of 3.0 ± 1.3 nM vs. 8.7 ± 3.9 nM of the NZO variant). These data suggest that resistance to the anorexigenic effect of NmU contributes to the obesity of NZO mice, and that this resistance may reflect an impaired signal transduction of the NZO NmuR2 variant (Schmolz et al. 2007).

## Candidate genes identified in outcross populations carrying the *ob* or *db* mutation

### Sorcs1

In obese mice of an intercross between C57BL/6J-*ob/ob* and BTBR T+tf/J mice (Clee et al. 2006), a major QTL on Chr 19 responsible for impaired insulin secretion and beta cell degradation was identified. Introgression of the chromosomal segment of B6 to the BTBR strain and analysis of a series of subcongenic lines defined a critical region of 242 kb. In this interval, *Sorcs1* was the only gene with amino acid substitutions of altered expression, and was thus considered the most likely candidate. SORCS1 binds platelet derived growth factor, and it was suggested that the gene has a role in expanding or maintaining the islet vasculature. SNPs in the human *SORCS1* gene were found to be associated with fasting insulin levels in the Mexican American Coronary Artery Diseases cohort, and with diabetes risk in women of the San Antonio Family Diabetes Study (Clee et al. 2006).

### Lisch-like

In the obese F2 progeny of a C57BL/6J-ob/+x DBA/2J-ob/+intercross, a QTL conferring diabetes (hyperglycaemia, hypoinsulinemia, disrupted islet morphology) was mapped to distal Chr 1 (Dokmanovic-Chouinard et al. 2008). The diabetogenic allele was contributed by the DBA/2 J background. A critical region of 1.8 Mb in the QTL comprising 14 genes was identified with a series of subcongenic lines. Of these, the gene *Lisch-like* was identified by mRNA expression analysis and sequencing as the most likely candidate. Knockdown of the gene in zebrafish produced a dispersed phenotype of insulin-producing cells. In addition, ENU-induced null mutation of *Lisch-like* produced a phenotype that was similar to that of the subcongenic lines (Dokmanovic-Chouinard et al. 2008). So far, the function of *Lisch-like* is completely unknown.

### Tomosyn-2

In the intercross employed to identify *Sorcs1* (see above), a second type 2 diabetes locus was identified on Chr 16 (Bhatnagar et al. 2011). Introgression of BTBR T+Tf/J Chr 16 into the B6 background generated consomic mice which exhibited elevated fasting glucose and lower insulin levels. A critical region of 1.6 Mb was determined with subcongenic lines. The second phase of insulin secretion was reduced in islets from congenic lines carrying the BTBR-derived chromosomal segment. The most likely candidate in the region was tomosyn-2 which exhibited a difference in expression and a non-synonymous SNP within the coding region. Overexpression of the BTBR variant of *Tomosyn-2* in INS1 cells inhibited insulin secretion. In addition, the B6 variant appeared more susceptible to proteasomal degradation (Bhatnagar et al. 2011). These data suggest a major functional difference between the variants which could be responsible for the diabetic phenotype.

### Tuberous sclerosis complex 2 *(Tsc2)*

In the C57BL/6J-ob/+x BTBR-ob/+intercross, a QTL for fatty liver was mapped to Chr 17 (Wang et al. 2012). As the most likely candidate gene responsible for this phenotype, *Tsc2* was identified. *Tsc2* is an inhibitor of the target of rapamycin, and is involved in cell growth and proliferation. Heterozygous *Tsc2* knockout mice exhibit an increased expression of lipogenic genes in the liver. The allelic difference between BTBR and B6 is a coding SNP which leads to an altered gene expression when transfected in AML12 cells, and an altered proliferation of Ins1 cells (Wang et al. 2012).

### Amyloid precursor protein (*App*)

A comprehensive analysis of the interactions between genotype, mRNA expression in 5 tissues, and plasma insulin in obese mice of the B6 × BTBR intercross (see above) led to the identification of the Alzheimer gene *App* as a candidate regulator of insulin secretion (Tu et al. 2012). *App* knockout mice exhibited increased insulin secretion in response to glucose, suggesting mechanistic similarities between the neurodegenerative disease and type 2 diabetes (Tu et al. 2012).

### Ubiquitin-conjugating enzyme E2L6 (*Ube2l6*)

The analysis of obese progeny of a (C57BL/6J *ob*/+ × BALB/c *ob*/+) × BALB/c ob/+ backcross population identified a BALB/c-derived QTL (*Lipq1*) on Chr 2 protecting against obesity by increased lipolysis and ATGL (adipocytes triglyceride lipase) expression in adipose tissue (Marcelin et al. 2012). By introgression of a segment of C57BL/6J Chr 2 to the BALB/c background, a critical interval of 9.8 Mbp was defined in which a nonsynonymous coding SNP in the gene encoding the ubiquitin-conjugating enzyme E2L6 (*Ube2l6*) was identified. The BALB/c allele of *Ubc2l6* is hypomorph, and its expression is markedly reduced in adipose tissue of BALB/c mice, presumably causing the decrease in adipocyte size and number. Accordingly, suppression of *Ube2l6* in 3T3L1 adipocytes led to a reduced triglyceride accumulation (Marcelin et al. 2013).

## Genes associated with alterations of glucose homeostasis identified in crosses between lean strains

### Solute carrier family 35 member B4 (*Slc35b4*)

In crosses between C57BL/6J and A/J mice, a QTL on Chr 6 was identified which was responsible for 65 % of the body weight difference between the parental strains. A chromosome 6 substitution strain was used to further dissect this QTL with congenic and subcongenic lines, and evidence for a complex architecture of 3 different loci modifying body weight was obtained (Yazbek et al. 2011). The subcongenic lines also indicated that the QTL was responsible for a differences in insulin sensitivity and glucose production between strains. In one of these loci, the solute carrier family 35, member B4 (*Slc35b4*) was identified as the most likely candidate. SLC35B4 is localized in the Golgi membrane and facilitates transport of UDP-xylose and UDP-N-acetylglucosamine into the Golgi apparatus. Although no DNA sequence variation was found, mRNA levels of *Slc35b4* were 1.5-fold higher in livers of subcongenic lines carrying the A/J allele. Knockdown of *Slc35b4* in liver cells suggested that the gene is involved in the regulation of glucose production by the liver (Yazbek et al. 2011).

### Amyloid P component, serum (*Apcs*)

In an intercross between C57BL/6J and C3H/HeJ (C3H) apoliporotein E-deficient mice, a QTL for fasting glucose was mapped to distal Chr 1 (Li et al. 2012). Generation of congenic strains that were challenged with a high-fat diet confirmed the QTL. A gene (*Apcs*) located in the peak region of the QTL was identified as a likely candidate because of an allelic difference in its expression in liver. *Apcs* encodes serum amyloid peptide, and protein levels in serum increased in response to the high-fat diet. In addition, transgenic expression of *Apcs* from C3H reduced glucose intolerance of B6.

### Nicotinamide nucleotide transhydrogenase (*Nnt*)

Genome wide linkage analysis of a F2 intercross of C57BL/6J × C3H/HeH identified a QTL on Chr 13 for impaired insulin secretion due to a failure of glucose to close K_ATP_ channels (Toye et al. 2005). The mitochondrial proton pump *Nnt* is located in the peak region of the QTL, and its expression was markedly lower in liver and islets of the B6 strain due to a deletion of exons 7–11. Direct evidence for the functional role of *Nnt* was obtained by siRNA knockdown in MIN6 cells which markedly reduced glucose-induced calcium influx and insulin secretion. Transgenic expression of the normal *Nnt* gene rescued the defective insulin secretion and glucose tolerance of B6 mice (Freeman et al. 2006). These data suggest that expression of a normally functioning NNT proton pump is required for adequate insulin secretion. However, two more recent studies reported contradictory results: Wong et al. (2010) compared *ob* B6 substrains with normal or disrupted *Nnt* and found no difference in glucose-stimulated insulin secretion or insulin sensitivity, whereas Simon et al. (2013) observed impaired glucose tolerance in B6/J versus B6/N mice.

## General conclusions and perspectives

The above summarized studies show that conventional positional cloning is a valid strategy to identify mouse genes that are responsible for obesity-associated diabetes. By the generation of subcongenic lines of a QTL, if possible starting with chromosome substitution strains, then small critical regions that harbor the gene(s) in question can be identified with certainty. Sequence analysis and mRNA profiling together with gene targeting in-vitro and in-vivo may lead to a solid chain of evidence linking sequence differences with altered molecular, cellular, and physiologic function, thereby establishing causality for a candidate gene. As far as the solidity of the evidence is concerned, the strategy appears superior to the genome wide association studies carried out in humans which, in the absence of direct functional evidence, leads to susceptibility loci only. However, it should be noted that not all of the published mouse candidate genes are based on irrefutable direct evidence, and it would not be surprising if some of them will turn out to represent false positives. Furthermore, with the mouse strains employed so far, only a part of the full complexity of diabesity may be accessible. Thus, the panel of recombinant strains (Collaborative Cross) derived from 8 founders which was generated by the Complex Trait Consortium might become an important research tool in the future.

A surprising result of the search for diabetes genes is that there is little overlap between mouse genes identified by positional cloning and human genes (loci) identified by genome-wide association studies. So far, only a few obesity mouse genes identified by GWAS overlap with human obesity candidates (Parks et al 2013). A potential reason for this limited overlap could be the difference in the degree of homozygosity between humans and laboratory mice. Mice have probably lost variants that produce severe phenotypes when both alleles are affected. This does not mean that results obtained from mouse models cannot be transferred to the human disease: Most of the above-described genes show relations with the human disease, e.g., by altered gene expression or by involvement in a pathway that is also connected with the human diabetes. Thus, mouse studies lead to pathways that may be disrupted in both humans and mice, and could thereby lead to novel therapeutic targets. As an example, the identification of the adipogenic/diabetogenic alleles of *Tbc1d1*, *Zfp69*, and *Ifi202b* supports the concept that fat oxidation and fat storage are crucial determinants of obesity and diabetes. Thus, the identification of mouse obesity and diabetes genes is a reasonable strategy to study the pathogenesis of both mouse and human disease.
